# Zika virus infection histories in brain development

**DOI:** 10.1242/dmm.050005

**Published:** 2023-07-17

**Authors:** Bruna L. M. Marcelino, Brendha L. dos Santos, Jhulimar G. Doerl, Samantha F. Cavalcante, Sara N. Maia, Nivia M. R. Arrais, Andrea Zin, Selma M. B. Jeronimo, Claudio Queiroz, Cecilia Hedin-Pereira, Eduardo B. Sequerra

**Affiliations:** ^1^ Brain Institute, Federal University of Rio Grande do Norte, 59056-450 Natal, Brazil; ^2^ Morphological Sciences Program, Institute of Biomedical Sciences, Federal University of Rio de Janeiro – Rio de Janeiro, 21941-590 Rio de Janeiro, Brazil; ^3^ Neurosciences Graduate Program, Federal University of Rio Grande do Norte, 59056-450 Natal, Brazil; ^4^ Department of Pediatrics, Onofre Lopes University Hospital, Federal University of Rio Grande do Norte, 59012-300 Natal, Brazil; ^5^ Clinical Research Unit, National Institute for Women's, Child and Adolescent Health Fernandes Figueira, Oswaldo Cruz Foundation, 59056-450 Rio de Janeiro, Brazil; ^6^ Institute of Tropical Medicine of Rio Grande do Norte, Federal University of Rio Grande do Norte, 59056-450 Natal, Brazil; ^7^ National Institute of Science and Technology of Tropical Diseases, Natal, Brazil; ^8^ Vice-Presidency of Research and Biological Collections (VPPCB), Oswaldo Cruz Foundation, 21040-900 Rio de Janeiro, Brazil; ^9^ National Institute for Science and Technology on Neuroimmunomodulation (INCT-NIM), Oswaldo Cruz Institute, Oswaldo Cruz Foundation, 21040-900 Rio de Janeiro, Brazil

**Keywords:** Susceptibility window, Calcification, Brainstem, Cerebellum, Blood–brain barrier, Neuronal migration

## Abstract

An outbreak of births of microcephalic patients in Brazil motivated multiple studies on this incident. The data left no doubt that infection by Zika virus (ZIKV) was the cause, and that this virus promotes reduction in neuron numbers and neuronal death. Analysis of patients' characteristics revealed additional aspects of the pathology alongside the decrease in neuronal number. Here, we review the data from human, molecular, cell and animal model studies attempting to build the natural history of ZIKV in the embryonic central nervous system (CNS). We discuss how identifying the timing of infection and the pathways through which ZIKV may infect and spread through the CNS can help explain the diversity of phenotypes found in congenital ZIKV syndrome (CZVS). We suggest that intraneuronal viral transport is the primary mechanism of ZIKV spread in the embryonic brain and is responsible for most cases of CZVS. According to this hypothesis, the viral transport through the blood–brain barrier and cerebrospinal fluid is responsible for more severe pathologies in which ZIKV-induced malformations occur along the entire anteroposterior CNS axis.

## Teaser

**Summary:** We discuss which mechanisms govern the embryonic susceptibility window for Zika virus infection, how congenitally infected patients' neurological disorders develop, and how developmental biology studies unveil the mechanisms of viral spread.

## Introduction

In 2015, Zika virus (ZIKV) emerged as a new pathogen that can be transmitted from the mother to the embryo/fetus (Calvet et al., 2016; Cugola et al., 2016). ZIKV is a mosquito-borne RNA arbovirus. Viral infection in adults causes fairly mild disease, characterized by fever, rash, headache, conjunctivitis, and joint and muscle pain, lasting a few days. However, if the infection occurs in pregnancy, ZIKV is vertically transmitted and disturbs the development of the embryonic or fetal nervous system. The range of brain abnormalities varies between patients, and can include microcephaly, cerebellar hypoplasia, lissencephaly, ventriculomegaly, brainstem dysfunction and brain calcifications. In surviving newborns, these lead to clinical symptoms that include intellectual disabilities, movement disorders and epilepsy. This group of phenotypes was named congenital ZIKV syndrome (CZVS; Aragao et al., 2016, 2017; França et al., 2016; Moore et al., 2017). Our understanding of the central nervous system (CNS) malformations in CZVS patients and their functional consequences remains incomplete, but it continues to grow as the surviving patients mature. This Review summarizes the different ZIKV pathways of entry and spread in the embryonic and fetal CNS. We discuss the origin of neuronal migration defects and calcifications and their timing during development. Lastly, we propose that different timings and pathways for ZIKV entry and spread in the embryo account for the diversity of CZVS phenotypes observed in patients.

## Susceptibility window for ZIKV infection of the developing CNS

Understanding the variety of phenotypes in CZVS requires identifying which developmental stages are susceptible to ZIKV. In this respect, most of the information we have about the time of congenital infection comes from either the medical records that indicate in which gestational week (GW) pregnant people reported symptoms or the timing of detection of the virus in bodily fluids. Analysis of a cohort of 244 patients in Rio de Janeiro revealed that CNS malformations occurred within a broad temporal window of maternal infection, ranging from the fifth to the 38th GW (Brasil et al., 2020). Our group also observed this temporal variance in the city of Natal, with reports showing a long possible infection window from the third to the 28th GW (Sequerra et al., 2020). These findings suggest that ZIKV can access the placenta and the embryo throughout the intrauterine period.

### ZIKV infection prior to neural induction

**Fig. 1.**
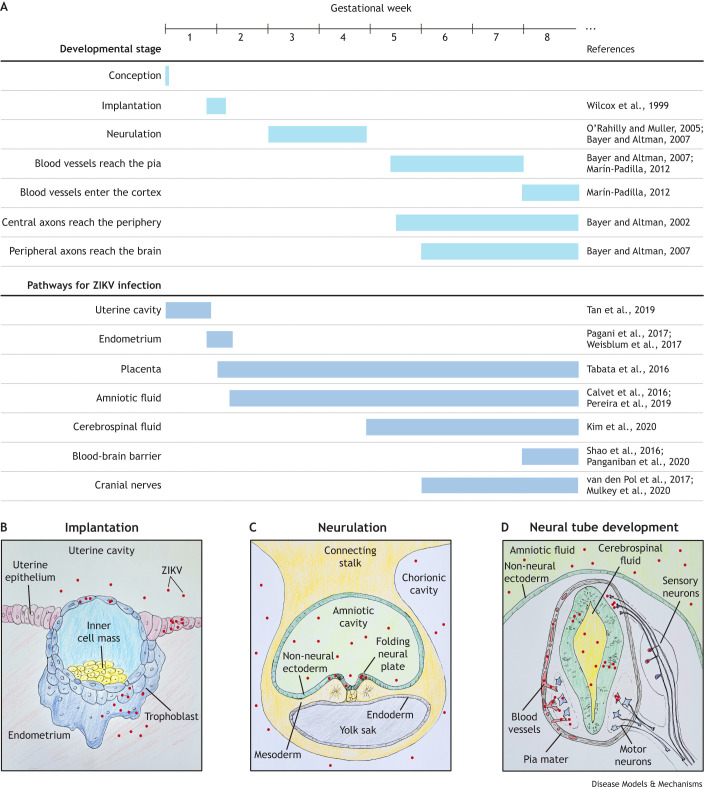
**Human early embryogenesis milestones and the possible routes for Zika virus (ZIKV) infection and spread throughout the central nervous system (CNS).** (A) Timeline of key human developmental milestones and the appearance of pathways for ZIKV transport to the embryonic CNS. Relevant references that support each milestone timing and infection pathway are provided in the right column. (B-D) Representative drawings of the development of routes for ZIKV transport in the early embryo. (B) Transverse section of the embryo implanting in the endometrium. After the blastocyst hatches from the zona pellucida, trophoblast cells come into direct contact with the uterine environment. ZIKV infects trophoblast cells and possibly reaches the embryonic cells in the inner cell mass. After the embryo implants in the endometrium, it loses its direct contact with the uterine cavity. However, ZIKV can infect endometrial cells. Therefore, ZIKV can infect trophoblast cells through the uterine cavity and through the endometrium. (C) Transverse section of the trilaminar embryonic disc surrounded by the amnion and yolk sac. Human neurulation starts at the third gestational week (GW). At this stage, neural cells are in contact with the amniotic fluid, which can also become infected. ZIKV in the amniotic fluid infects the neural plate cells but not the non-neural ectoderm. It does not display tropism for mesoderm or endoderm cells either. (D) Transverse section of the neural tube at the second month of gestation. After the non-neural ectoderm closes, the amniotic fluid is separated from the neural tube and is therefore not a possible direct infection pathway anymore. From this stage on, to infect the CNS, ZIKV has to cross through the barriers between the blood and the meninges (represented on the left side of the neural tube in red and gray, respectively), the blood and the cerebrospinal fluid, or the blood and the brain parenchyma (represented on the left side of the neural tube). Alternatively, ZIKV needs to be transported along the nerves that leave (motor neurons; blue) or enter (sensory neurons; purple) (both neuron types are represented on the right side of the neural tube) the CNS.

In the earliest phases of development, the embryo is in the oviduct. It is protected by the zona pellucida, a specialized mesh-like extracellular matrix that, aside from regulating fertilization, provides a barrier to the penetration of different viruses (Van Soom et al., 2010). However, the protective effect of the human zona pellucida against ZIKV infection is unknown. After the blastula hatches, the trophoblast, a layer of cells that attach to the endometrium and differentiate in part of the placenta, comes into contact with the uterine cavity. The trophoblast remains exposed to uterine fluids until implantation is complete (Fig. 1A,B). It is not clear whether ZIKV reaches the uterine cavity after infection via a mosquito bite, but ZIKV infection at these early stages increases the risk of pregnancy loss and poor intrauterine growth. Tan and colleagues demonstrated that ZIKV can infect human and murine trophoblast cells and induce apoptosis (Tan et al., 2019). Moreover, infection of immunosuppressed pregnant mice at the preimplantation stage of the pregnancy led to a lower recovery of blastocysts by uterine flushing, and half of them were infected. This infection correlated with miscarriages, with a minority of pregnancies of ZIKV-infected mice proceeding to fetus formation (Tan et al., 2019). These data, therefore, indicate that circulating ZIKV can reach the uterine tract, at least in immunosuppressed mice, and infect the pre-implanted embryo, either leading to its loss or to detrimental effects on further development.

Furthermore, the infected endometrium may also be a source of embryo infection at implantation (Fig. 1A,B). *In vitro* differentiation of an immortalized endometrial cell line into a decidua-like phenotype increases the expression of the cellular ZIKV receptors AXL and MER (MERTK), and subsequent viral replication in the decidua-like cells (Pagani et al., 2017). Similarly, decidua explants from the first trimester of pregnancy can be infected by ZIKV and permit virus replication (Weisblum et al., 2017).

How do the neural progenitors in the embryo become infected when maternal infection occurs in the first weeks of gestation? Does the ZIKV-infected inner cell mass of the blastocyst progress through gastrulation until neural induction? To hopefully answer these questions, Tan and collaborators analyzed embryonic day (E)10.5 embryos (corresponding to neural tube development at the fourth to fifth GW in humans) of ZIKV-infected immunosuppressed mice that survived infection at pre- and peri-implantation stages of gestation (Tan et al., 2019). These embryos expressed ZIKV antigens in their heads. Nevertheless, these embryos could also have been infected after neurulation, because the pregnant dams displayed persistent infection in their brains and spleens when the embryos were harvested (Tan et al., 2019). An important caveat is that this study used immunosuppressed animals. The maternal infection could have persisted due to the impaired immune response that prevented the dams from clearing the virus. However, because immunocompetent mice do not develop a ZIKV viremia, different groups opted for this method for modeling vertical transmission in mice (Box 1).

**Box 1. Murine models for the study of congenital Zika virus (ZIKV) infection**

The mouse is an important model for studying neural development. After the outbreak of births of microcephalic patients due to ZIKV infection, multiple groups have attempted to reproduce congenital ZIKV syndrome (CZVS) in these animals. Their studies have used different mouse lines and infection doses, times and routes to achieve that. When choosing a murine strain for modeling CZVS, researchers must consider several factors, from its fecundity, suitability for neuroscience research and immune status to amenability for genetic manipulation.

In the first attempt to demonstrate vertical transmission of ZIKV from mother to fetus in mice, Cugola and colleagues intravenously injected pregnant C57BL/6 and SJL mice with various ZIKV titers on days 10-13 of gestation, which corresponds to early neurogenesis in the embryos (Cugola et al., 2016). The choice of mouse strain is important for such studies – C57BL/6 mice are inbred and one of the most widely used murine strains for disease modeling and neuroscience research (Simon et al., 2013), whereas SJL mice are a less prevalent inbred strain that displays different immune responses to those of other mouse lines, which change their susceptibility to different viruses (Matsushima and Stohlman, 1990). Cugola and colleagues showed that high-titer ZIKV infection did not lead to embryo infection or morphological abnormalities in C57BL/6 mice, but SJL embryos displayed growth restriction, brain infection and increased neural cell death, and pregnant SJL dams had prolonged ZIKV viremia compared to that of C57BL/6 dams (Cugola et al., 2016; Shiryaev et al., 2017). It is unknown why adult SJL mice develop this prolonged ZIKV viremia. However, separate studies have demonstrated that SJL mice are more susceptible to neurological complications caused by Theiler's murine encephalomyelitis virus (Stewart et al., 2010) and display a weaker and slower immune response to it (Ciurkiewicz et al., 2021). It is therefore possible that the immune status of SJL has an important role in the embryos' susceptibility to ZIKV. Therefore, genetic differences between murine lines can determine the susceptibility of adult mice and embryos to ZIKV, which can be helpful for understanding the differences in susceptibility found between human populations.

Although C57BL/6 adult mice do not develop ZIKV viremia, this mouse line is amenable to genetic manipulation. Researchers have used this genetic background to knock out various genes related to type I interferon immunological response. *Ifnar1* single knockout, *Ifnar1/2* double knockout, and *Irf3*, *Irf5* and *Irf7* triple knockout lines are all artificially susceptible to ZIKV infection, which causes high mortality in adult knockout animals (Morrison and Diamond, 2017). Using these transgenic lines allows the experimenter to model vertical transmission of ZIKV from the mother to the fetus, but, because the animals are immunosuppressed, infection induces severe maternal distress, which is not common in humans. Interestingly, crossing *Ifnar1* knockout females with wild-type males improves the growth of ZIKV-infected embryos when infection occurs during cortical neurogenesis. Congenital infection in earlier phases of gestation, however, kills the embryos (Chen et al., 2017). Therefore, type I interferon response is fundamental for the maternal immune reaction that prevents vertical transmission in mice.

Although adult C57BL/6 mice do not develop ZIKV viremia, the embryos and the tissues of the maternal–fetal interface may not be as resistant. Based on that, two groups developed strategies to model vertical ZIKV transmission in C57BL/6 mice without developing viremia in the pregnant dams (Xavier-Neto et al., 2017; Pedrosa et al., 2020). These strategies are based on the idea that the placenta is an immune-privileged organ and therefore more susceptible to viral infection than other maternal organs. Both groups were able to analyze the effect of infection at different developmental stages. ZIKV injection in the jugular vein of the pregnant dam produced vertical transmission because it gave the virus reasonably quick access to the placenta, before the dam's immune system eliminated it from the blood (Xavier-Neto et al., 2017). Moreover, injecting the viral suspension into the bloodstream recapitulates the natural way in which ZIKV infects humans, in which the virus enters the bloodstream via a mosquito bite. An alternative approach is the injection of ZIKV into the peritoneal cavity. Intraperitoneal injection of pregnant dams at embryonic day 12, which is when early cortical neurogenesis occurs in the embryos, produces an embryonic brain infection that lasts at least 8 days (Pedrosa et al., 2020). The advantage of these intravenous or intraperitoneal maternal infection strategies is that they do not require surgery to access the embryos. However, both remain to be replicated by other groups.

Although infecting pregnant dams to trigger vertical transmission is a technically easy approach, a different way to study ZIKV infection during development is by directly infecting embryos or newborn pups. These experiments need to be timed so that the pups become infected before their immune system matures. Embryos are usually injected in the lateral ventricle, while postnatal pups can also be injected subcutaneously or intraperitoneally. Immunocompetent C57BL/6, Kunming and ICR juvenile mice are susceptible to developing a fatal disease when receiving an intraperitoneal ZIKV injection, whereas BalB/c pups do not produce any signs of disease (Li et al., 2018). Intraperitoneal injection of ZIKV in C57BL/6 mice at different ages reveals that the window of susceptibility lasts for the first two postnatal weeks and is closed at postnatal day 14 (Li et al., 2018). In terms of cerebral cortex development, the first postnatal week of the mouse is equivalent to the second trimester of human gestation (Bayer and Altman, 2007). Therefore, mice have to feed and hydrate on milk at the same stage of brain development as that when humans still receive nutrients through the placenta. Such differences can explain the high mortality of ZIKV infection in newborn mice, which we do not observe in human fetuses.

These results are just a brief summary of the extensive body of work in the literature. For space constraints, we were not able to discuss all the valuable contributions to the field of pregnant mice experiments with ZIKV. However, adapting the variables discussed here can lead to better reproduction of the phenotypes found in CZVS. At the same time, it is important to acknowledge fundamental differences, such as developmental speed, placenta characteristics, developmental maturity at birth and others, that limit the array of answers that mouse experiments can provide. Complementary models, such as *in vitro* and *ex vivo* human tissue cultures, organoids and non-human primates, are available and can help answer the key open questions in CZVS research.

There are ways of inducing vertical ZIKV transmission in mice in the absence of viremia. Xavier-Neto and collaborators developed such a model by injecting ZIKV into the jugular vein of immunocompetent pregnant mice (Xavier-Neto et al., 2017). The authors injected dams at different stages of pregnancy, from gastrulation and neurulation to early fetal brain development. ZIKV exposure during gastrulation (broadly corresponding to the third GW in humans) led to a persistent infection in most dams and had pronounced teratogenic effects on the embryos. However, injection at a later stage of pregnancy, at E12.5, did not produce noticeable teratogenic effects. Most pregnant dams cleared the virus from their blood very fast, although the infection persisted in the spleens and livers of some dams (Xavier-Neto et al., 2017). These data suggest that the mouse placenta is more permeable to the fast, viremia-independent, ZIKV vertical transmission during gastrulation than at later stages of pregnancy.

The studies above provide a wealth of information on how maternal ZIKV infection affects the early embryo. However, there is no evidence that murine embryos that become infected with ZIKV prior to neural induction sustain neural development until they reach a developmental stage when there is a CNS.

Studies in non-human primates and humans suggest that the virus persists in the pregnant female and can reach the embryo days or weeks after the initial blood infection, meaning that the virus may reach the embryo after neural development has begun. Two studies report ZIKV persistence after experimental infection of pregnant rhesus monkeys. Gestation in these monkeys lasts ∼23 weeks (or 160 days). Nguyen and collaborators inoculated four monkeys at 31, 38 (early CNS development, closed neural tube), 103 and 118 days of gestation (comparable to neural development during the late second trimester in humans) (Nguyen et al., 2017). They observed that the two monkeys inoculated early in gestation, and one of the animals injected later, maintained the plasma viral RNA for at least 1 month. By contrast, non-pregnant animals cleared it within 15 days after infection (Nguyen et al., 2017). Martinot and collaborators inoculated six pregnant rhesus monkeys that were between the sixth and seventh GWs, and three animals that were between the 12th and 14th GWs (Martinot et al., 2018). The monkeys also maintained ZIKV RNA for a long time in their plasma, particularly those infected earlier in pregnancy. This prolonged infection in pregnant rhesus monkeys recapitulates well the viremia observed in humans.

Indeed, pregnant people also display prolonged ZIKV viremias. Driggers and collaborators reported a case of an individual infected at the 11th GW that electively terminated the pregnancy at the 21st GW. ZIKV RNA was detected in the extraembryonic membranes, amniotic fluid and maternal plasma, and in fetal tissues such as the brain, muscles and spleen (Driggers et al., 2016). In another case, a patient reported symptoms at the 10th GW. One week later, the fetus died, and the pregnancy was interrupted at the 13th GW. At this time, ZIKV RNA was present in the amniotic fluid, placenta, maternal serum and fetus (van der Eijk et al., 2016). Meaney-Delman and collaborators described four cases of patients who reported ZIKV infection symptoms in their 12th, 18th, 19th and 20th GWs, and a fifth asymptomatic individual who tested positive and got infected during the first 20 GWs (Meaney-Delman et al., 2016). One of these symptomatic patients elected to terminate the pregnancy at the 18th GW, 6 weeks after the symptoms. The fetus had a small head circumference, and viral RNA was present in fetal muscle and bone, as well as in the amniotic fluid and placenta. The other three symptomatic patients delivered healthy, possibly uninfected infants, but the maternal plasma remained positive for ZIKV RNA for at least 44, 46 and 53 days, respectively (Meaney-Delman et al., 2016). It is, therefore, possible that vertical transmission happens days to weeks after the maternal infection. Moreover, a prolonged infection can occur whether symptoms are present or not and is not a guarantee of vertical transmission.

The amniotic fluid can also function as a reservoir for viral transmission later in pregnancy. An initial study showed persistent ZIKV RNA in the amniotic fluid of two patients carrying fetuses with microcephaly, which was detected by ultrasound 10 and 18 weeks after the initial ZIKV infection symptoms (Calvet et al., 2016). A later study included nine patients whose fetuses had abnormal CNS ultrasound findings and a CZVS diagnosis. Seven of these tested positive for ZIKV RNA in their amniotic fluid, in intervals ranging from 10 to 27 weeks after the onset of symptoms (Pereira et al., 2019). Pregnancy appears to increase susceptibility to long viremias and persistence of tissue infection. Therefore, we must consider that if ZIKV infection occurs before the third GW, the virus could hypothetically remain incubated in the body for a long time before reaching the neural tube and triggering the cascade that eventually causes CZVS.

### ZIKV infection after the initiation of neural tube formation

Considering that ZIKV most likely infects the embryonic CNS after neural induction/neural plate differentiation, how early can the neural plate become infected and still sustain development until a live birth? The neural plate is in direct contact with the amniotic fluid until neural tube closure (Fig. 1C). The anterior neuropore closes during the third GW (Bayer and Altman, 2007), and the posterior neuropore closes at the end of the fourth GW (O'Rahilly and Muller, 2005; Fig. 1A). After closure, the non-neural ectoderm stays in contact with the amniotic fluid, forming a barrier that prevents direct contact with the neural tube, and the amniotic fluid that becomes trapped inside the closed neural tube forms the first cerebrospinal fluid (Fig. 1D). This developmental landmark is essential because if the amniotic fluid becomes infected before neural tube closure, ZIKV can infect the embryonic CNS independently from other pathways, such as through the blood vessels and nerves (Fig. 1A,D).

Blood infection of pregnant immunocompetent mice at E5.5 leads to neural tube defects in a subset of embryos (Xavier-Neto et al., 2017). However, at the time of writing, only one living patient has been documented to display a neural tube defect upon congenital ZIKV infection. This patient had myelomeningocele associated with microcephaly and demonstrated a persistent ZIKV infection in the cerebrospinal fluid for at least 43 days after birth (Contreras-Capetillo et al., 2018). Thus, early-pregnancy ZIKV infections in humans likely lead to embryo loss or resorption and may not be responsible for the phenotypes we observe in live births. Therefore, CZVS patients were probably CNS infected starting in the second month of gestation, after neural tube closure (Fig. 1A). Thus, we hypothesize that this stage of development represents the susceptibility window for CZVS. However, this does not necessarily mean that maternal ZIKV infection, or the onset of maternal symptoms, must occur at this stage. As discussed above, the virus can persist for a long time before reaching the fetus.

**Fig. 2.**
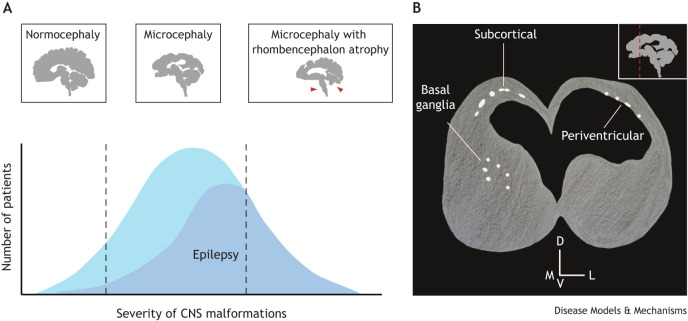
**The diversity of congenital ZIKV syndrome (CZVS) brain phenotypes and the relationship between anatomical and physiological outcomes.** (A) The severity of brain malformations in CZVS can be broadly divided into three types: (1) normocephaly with punctual malformations in the prosencephalon, (2) microcephaly with malformations restricted to the prosencephalon, and (3) microcephaly with caudal malformations (red arrowheads). The graph shows the approximate frequency of CZVS and CZVS-linked epilepsy cases across the brain malformation severity spectrum. Patients with caudal malformations have high odds of developing epilepsy, whereas normocephalic ones are less likely to be affected. (B) Schematic illustration of a coronal section of the brain of a postnatal CZVS patient as seen in a computed tomography examination. The inset in the top right corner represents a sagittal section of the brain, and the dashed red line indicates the position of the coronal section. Calcifications are represented in white. Subcortical calcifications are the most common in CZVS, but periventricular ones and calcifications in the basal ganglia also occur. D, dorsal; V, ventral; M, medial; L, lateral.

Although congenital brain malformations in CZVS (Aragao et al., 2016; 2017; Hazin et al., 2016; Moura da Silva et al., 2016; Rocha et al., 2016; Soares de Oliveira-Szejnfeld et al., 2016; Parra-Saavedra et al., 2017; Fig. 2) are the best-known sequelae of ZIKV neurotropism, the virus can also infect the adult nervous system. However, this infection has different features to those of the congenital one. Adults more often develop a peripheral nervous system syndrome similar to Guillain–Barré syndrome (Machado-Alba et al., 2016; Parra et al., 2016) but can, more rarely, develop CNS infections affecting the spinal cord (Mécharles et al., 2016) and the brain (Carteaux et al., 2016). These discrepancies suggest a different natural history for ZIKV infection in the CNS before or after birth. Because postnatal infections via mosquito bites cause transient symptoms in infants and no microcephaly or other CZVS phenotypes (Ramond et al., 2020), the data suggest that access of ZIKV to the CNS after birth occurs rarely (Mécharles et al., 2016; Carteaux et al., 2016).

However, subcutaneous injection of ZIKV in postnatal immunocompetent mice shows that systemic infection can culminate in CNS infection, leading to ventriculomegaly and seizures (Nem de Oliveira Souza et al., 2018; Ireland et al., 2020), which are otherwise typical of CZVS. One can interpret this as evidence that the window for CNS infection stays open after birth. However, unlike humans, postnatal mice are still completing the cortical neuronal migration and differentiation (Hoshiba et al., 2016). In humans, this process completes around the 24th GW (de Azevedo et al., 1997; 2003; Kostović and Jovanov-Milošević, 2006). Therefore, the immunological activity, CNS barriers and other features in neonatal mice are not equivalent to those in neonatal humans and represent different constraints for ZIKV.

Although birth seems to close the window for CZVS, it does not necessarily represent the end of ZIKV activity in the newborn's CNS. A small group of microcephalic patients diagnosed with CZVS showed progressive enlargement of the ventricles and loss of brain parenchyma after birth (Jucá et al., 2018; van der Linden et al., 2019). This enlargement was associated with the development of hypertensive hydrocephalus. van der Linden et al. (2019) reported that the cerebrospinal fluid of these patients did not have detectable ZIKV. Thus, it is more probable that the worsening brain malformations are not caused by viral persistence but by continuous inflammation. Most of the mothers of the patients that developed postnatal hydrocephalus reported ZIKV infection symptoms in the second trimester of pregnancy (van der Linden et al., 2019), indicating a long interval between maternal infection and worsening brain phenotype in the infants. Even milder cases on the CZVS spectrum can develop into more severe phenotypes. Some CZVS patients that are normocephalic at birth develop secondary microcephaly that is only detected postnatally (Nielsen-Saines et al., 2019; Siqueira Mello et al., 2019).

## Neuronal migration and epileptogenesis in CZVS

One of the main determinants of the low quality of life of CZVS patients and their families is the occurrence of spontaneous seizures. Epilepsy secondary to congenital viral infection is observed not only in CZVS but also after congenital infection with cytomegalovirus, herpes, Epstein-Barr and varicella-zoster virus (Bartolini et al., 2019; Zhang et al., 2020; Wouk et al., 2021). Despite significant alterations to brain morphology, not all CZVS patients develop recurrent and spontaneous seizures. The prevalence of epilepsy in CZVS patients varies, ranging from 34% to 79% in the first year of life and increasing as the patients age (Maia et al., 2021). Moreover, seizure severity and semiology vary significantly. This heterogeneity means that ZIKV infection is necessary but may not be sufficient for developing unprovoked seizures later in life. Although brain malformations alone are not enough to cause epilepsy, one should expect correlations between brain anatomical features and seizures. Cerebellar and brainstem atrophy positively correlate with seizure occurrence (Fig. 2A). Likewise, smaller prosencephalon volumes correlate with higher rates of pathological electrical activity that neurons generate between seizures (interictal spikes; Sequerra et al., 2020). In addition, calcifications (a common feature of CZVS that we describe in more detail below) around the ventricles or in the ventral prosencephalon (Fig. 2B), particularly the basal ganglia and thalamus, increase the odds of an epilepsy diagnosis in CZVS patients with microcephaly (Sequerra et al., 2020). Surprisingly, normocephalic CZVS patients can also develop epilepsy, but at a much lower rate than do microcephalic patients (Blackmon et al., 2020; Fig. 2A).

Epilepsy syndromes that arise after congenital or early-life viral infection could result from immune (Zhang et al., 2020) and neural responses to the infection. Inflammation or inflammatory cytokines can change the excitability properties of neurons (Smeal et al., 2012; Libbey et al., 2016; Cusick et al., 2017), and cellular and synaptic reorganization disturbances correlate with widespread cell death and scar formation (sclerosis). Here, we hypothesize that congenital ZIKV infection and the subsequent inflammatory response impact the generation and migration of excitatory and inhibitory neurons, disturbing their equilibrium and resulting in seizure activity.

**Fig. 3.**
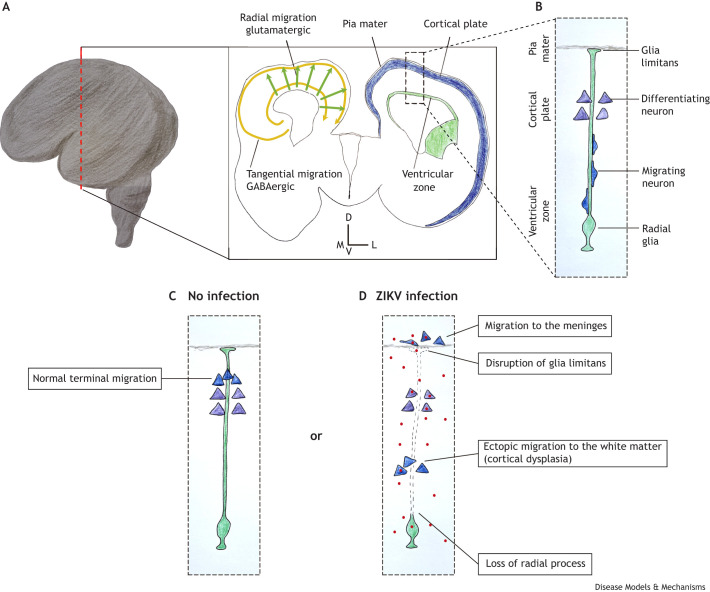
**ZIKV-induced neuronal migration defects.** (A) Schematic representation of a human brain at the 17th GW. The dashed red line indicates the position of the coronal section represented in the rectangle on the right. The schematic of the left hemisphere shows the two types of neuronal migration in the cortical plate: the tangential migration of inhibitory GABAergic neurons (yellow arrows) and the radial migration of excitatory glutamatergic neurons (green arrows). In the schematic of the right hemisphere, the proliferative layers are represented in green and the developing cortical plate is depicted in blue. The dashed line box indicates the localization of panels B-D. (B) At this stage of brain development (17th GW), radial glia form a physical support for the migration of newly generated neurons. The schematic shows that the neurons that previously migrated and are starting to differentiate reside closer to the basal process of the radial glia (where it attaches to the pia mater). (C) In non-infected developing brains, the migrating neurons pass through the previously formed cortical layers and detach from the radial glia closer to the pia, clustering in the cortical plate. (D) In ZIKV infection, the basal processes of radial glia can become deformed or are lost, diminishing cues for proper neuronal migration. Moreover, the loss of glia limitans (the radial glial foot in the pia) increases the ‘permeability’ of the zone between the brain parenchyma and the meninges, allowing developing neurons to ectopically migrate to the meninges. Additionally, loss of radial glia processes causes premature interruption of neuronal migration, resulting in ectopic accumulation in the white matter. D, dorsal; V, ventral; M, medial; L, lateral.

During embryogenesis, neural progenitors receive signals that instruct them to differentiate into specific neuronal types depending on their position along the neural tube axes (Gunhaga et al., 2000; 2003). This positional information leads to the generation of neurons expressing different neurotransmitters. For instance, progenitors in the dorsal telencephalon generate excitatory glutamatergic neurons (Gorski et al., 2002), whereas progenitors in the ventral telencephalon generate inhibitory GABAergic neurons (Anderson et al., 1997; Wichterle et al., 2001; Al-Jaberi et al., 2015; Fig. 3A). The distance between the site of origin of excitatory and inhibitory neurons affects how these two populations reach their final position. Cortical excitatory neurons migrate from the underlying ventricular zone to the forming cortical plate. By contrast, inhibitory neurons migrate long distances for an extended period (Marín and Rubenstein, 2001; Fig. 3A). During neural circuit development, it is crucial that the correct number of these two types of neurons reach structures like the cerebral cortex and hippocampus, produce the right number of synapses, and tune the level of excitability and inhibition in the forming network (Joseph and Turrigiano, 2017). Corroborating this, mice lacking *Dlx1*, which encodes a transcription factor involved in the differentiation and survival of inhibitory neurons, have fewer subtypes of cortical and hippocampal inhibitory neurons than control mice, and develop epilepsy (Cobos et al., 2005). Moreover, neuronal migration defects, either caused by a loss-of-function mutation of the murine *Reln* or a splicing defect in its human homolog, cause higher susceptibility to epilepsy (Hong et al., 2000; Patrylo et al., 2006). These results point towards migration as a significant developmental event that may be perturbed in CZVS patients with epilepsy.

In the developing CNS, progenitor cells are closer to the ventricles, while newborn neurons and astrocytes migrate towards the pia mater to form the gray matter (Bystron et al., 2008; Fig. 3A-C). Evidence that ZIKV infection affects neuronal migration in CZVS comes from histopathological analyses of the brains of deceased patients. These show glia limitans malformation and the consequent abnormal migration of neurons and glia to the meninges (Fig. 3D); cortical dysplasia, when neurons terminally differentiate with incorrect placement (Fig. 3D); and macroscopic observations such as polymicrogyria or lissencephaly (Acosta-Reyes et al., 2017; Chimelli et al., 2017). These visible abnormalities are also common in genetic syndromes with defective neuronal migration (Feng and Walsh, 2001), but infection-derived malformations can be multifactorial. Analysis of genome methylation of blood samples from microcephalic CZVS patients demonstrated that the promoters of several genes involved in the regulation of neuronal migration and axonal targeting are hypermethylated (Anderson et al., 2021), meaning that the expression of these genes is downregulated. Therefore, the phenotypes and molecular evidence found postnatally indicate that the disruption of neuronal migration contributes to brain malformations occurring after congenital ZIKV infection.

There are not many studies on the impact of ZIKV infection on neuronal migration in animal models. Infection of *ex vivo* mouse telencephalic slices with ZIKV results in the accumulation of neurons closer to the ventricles (Rosenfeld et al., 2017; Fig. 3D), whereas normally they should migrate towards the pia mater. The accumulation of glutamatergic excitatory neurons in the subcortical region, closer to the ventricles, is associated with epilepsy (Crino, 2015). The effect of ZIKV infection on the migration of GABAergic neurons is unknown.

It is unclear whether ZIKV infection of young neurons directly affects their migration, but data suggest that this virus affects the environmental cues that guide neurons to their final position. In particular, newborn excitatory cortical neurons use radial glia (RG) processes as guides in their migration to the cortical plate (Rakic, 1972; Fig. 3B), which may be affected by ZIKV. The bodies of RG reside in the proliferative zone close to the ventricles and extend long processes that attach to the pia mater (Fig. 3B), and the effects of ZIKV infection on RG processes have been studied in various *in vivo* and *in vitro* models and human postmortem tissues. In a mid-gestational olive baboon fetus, ZIKV infection caused defects in RG fibers and impeded the migration of cortical neurons (Gurung et al., 2019; Fig. 3D). Similarly, the aforementioned study by Rosenfeld and colleagues demonstrated that ZIKV perturbs the basal projections of RG, contributing to disturbed neuronal migration in *ex vivo* mouse telencephalic slices (Rosenfeld et al., 2017). Recapitulating the results in murine brain slices, ZIKV infection of postmortem human embryonic telencephalic slices disrupted the previously typical RG scaffold as early as 3.5 days after infection (Onorati et al., 2016). ZIKV also induces cell death in human brain organoids in the first few days postinfection. At day 12 postinfection, ZIKV-infected organoids were significantly smaller, and contained fewer and smaller ventricular zone-like structures, than their mock-infected counterparts. The remaining RG within the ZIKV-infected organoids maintained basic architectural features, such as the location of N-cadherin on the apical side (Krenn et al., 2021). However, these *in vitro* results do not recapitulate the *in vivo* work in mice and non-human primates discussed above, which show that RG guidance properties are affected in the long term upon ZIKV infection (Rosenfeld et al., 2017; Gurung et al., 2019).

In humans, congenital ZIKV infection alters the expression of genes necessary for histological integrity and neuronal migration. Aguiar et al. (2020) sequenced RNA from brain tissue of human neonates with CZVS that died soon after birth (ranging from 30 to 41 GWs) and demonstrated decreased levels of transcripts encoding collagen and increased levels of cell adhesion factors, which correlated with neuronal migration defects. It is important to note that, in this cohort, the intervals between maternal symptoms and the date of birth comprised many weeks, with some mothers reporting ZIKV symptoms as early as the first trimester of pregnancy (Aguiar et al., 2020). Taken together, these extensive *in vitro*, *in vivo* and clinical studies indicate that the spectrum of phenotypes seen in CZVS, including epilepsies, probably involve neuronal migration defects. However, disrupted neuronal architecture is not the only brain abnormality in CZVS, as brain-imaging studies of patients often reveal calcifications. Calcifications are particularly relevant to this Review, as CZVS-induced epilepsy can simultaneously be the cause and a consequence of this structural abnormality.

## Calcifications: their formation, position and significance in CZVS

One of the main radiological findings in CZVS patients is the accumulation of calcium salts in the brain. These accumulations, referred to in clinical practice as intracranial calcifications, can arise in the developing brain through two primary mechanisms: the uncontrolled entry of calcium into dying cells, termed dystrophic calcifications, and the release of calcium salts from blood vessels when the blood–brain barrier (BBB) integrity is compromised, termed vascular calcifications (McCartney and Squier, 2014). Although ZIKV preferentially infects neural stem cells and progenitors (Wu et al., 2016), only a minority of patients develop calcifications in the proliferative zones (Fig. 2B). This lack of ventricular calcifications can be due to the mechanisms of viral infection and spread through the BBB, brain parenchyma and cerebrospinal fluid. Postmortem studies of CZVS patients' brains report dystrophic calcifications only, including calcium granules in cells and neurites (Mlakar et al., 2016; Sousa et al., 2017; Dávila-Castrodad et al., 2018). This indicates that the leading cause of intracranial calcifications in these patients is the loss of membrane integrity in dying cells. Follow-up brain imaging of microcephalic CZVS patients revealed that the number, size and density of calcifications diminish after 1 year of life. Despite this decrease after birth, the remaining calcifications persist until at least 3 years of age (Alves et al., 2021), suggesting that some can be permanent.

One must remember that the published pathology studies of CZVS have relied on small groups of patients. This means that the possibility of vascular calcifications in this syndrome cannot be excluded, particularly because there is evidence that ZIKV infection can disturb the BBB. ZIKV injection into the blood of pregnant immunosuppressed mice (Garcez et al., 2018) or directly into the brain of mouse embryos (Shao et al., 2016) during early corticogenesis disrupts or delays the development of blood vessels in the cerebral cortex and retina, also altering brain permeability (Shao et al., 2016). As reviewed elsewhere (Mustafá et al., 2019), data from cellular and animal models demonstrate that ZIKV infects the endothelial cells of the BBB during acute viremia and crosses them without disrupting the barrier. However, after the infection becomes chronic, the resulting inflammation affects the BBB. ZIKV-infected adult rhesus monkeys exhibit BBB disruption, perivascular neuroinflammation and increased migration of lymphocytes (Panganiban et al., 2020). These results underscore the possibility of vascular calcifications forming alongside dystrophic ones in ZIKV-infected brains.

Can the location of intracranial calcifications in CZVS patients tell us about the origin of ZIKV infection? Some authors suggested that calcifications in the cortical–subcortical transition areas (Fig. 2B) of CZVS patients' brains are related to vascular dysfunction (Niemeyer et al., 2020). However, as discussed above, the existing clinical data do not support this hypothesis. Alternatively, the position of calcifications can reflect ZIKV's preference for infecting specific cell types. The rationale that viral tropism for specific cell types determines the localization of calcifications comes from studies on microcephaly upon congenital cytomegalovirus infection. In these cases, calcifications most commonly present near the ventricles (Bale et al., 1985), which corresponds very well with the location of neural progenitors and stem cells, which are permissive to cytomegalovirus infection (Kawasaki and Tsutsui, 2003; Kawasaki et al., 2017; Sun et al., 2020). The pattern of calcifications observed in CZVS is distinct from that in cytomegalovirus-induced microcephaly. The calcifications caused by ZIKV infection locate more often in the white–gray matter junction and in the nuclei of the basal ganglia and thalamus (Soares de Oliveira-Szejnfeld et al., 2016; Fig. 2B). Retallack and colleagues used cortical slices from post-mortem human fetal brains to demonstrate that *ex vivo* ZIKV infection preferentially clusters in the proliferative regions closer to the ventricles at 13-14 GWs, whereas this preference is lost in infected cortical slices derived from fetuses that were non-viable at 20-22 GWs (Retallack et al., 2016). At this later developmental stage, newly differentiating astrocytes populate the tissue between the ventricular zone and the cortical plate, and these astrocytes are prone to ZIKV infection, leading to a uniform distribution of infected cells throughout the tissue (Retallack et al., 2016). Accordingly, upon intraperitoneal injection in pregnant mice, ZIKV preferentially targets neural progenitors and astrocytes (Wu et al., 2016). Therefore, it is likely that if ZIKV infects fetal brains before or after RG differentiate into astrocytes, which starts around 22 GWs (de Azevedo et al., 2003), the infection will display different spatial distribution patterns and, consequently, specific localization of calcifications.

Beyond the initial preference for infecting glia and neural progenitors (Retallack et al., 2016; Wu et al., 2016), prolonged ZIKV infections permit viral entrance in neurons, which can also affect the formation and localization of calcifications. Experiments using human brain organoids show that neural progenitors and astrocytes become infected faster, but neurons are also permissive to ZIKV infection (Qian et al., 2016; Watanabe et al., 2017). When newborn mouse pups receive an intraperitoneal injection of ZIKV, their brains first display a predominantly glial infection pattern. A few days later, neuronal infection substitutes the glial (van den Pol et al., 2017). Upon infection of neurons, the virus spreads through dendrites and axons, including the commissural neurons that connect brain hemispheres and the optic nerve. These ZIKV-infected commissural neurons then pass the virus to other neurons in the contralateral cortex. Moreover, the infection of ganglion cells in the retina leads to ZIKV transport to the visual thalamus (van den Pol et al., 2017). Together with the pathological evidence, these data suggest that the pattern of calcifications observed in the junction between the gray and white matter reflects the loss of calcium permeability control in neurons. Because ZIKV leads to neuronal accumulation in this junction (see above; Fig. 3D), we hypothesize that many displaced neurons die and contribute to calcification. Therefore, we propose that the pathways through which ZIKV spreads work together with its cell-type tropism in determining the spectrum of malformations seen in CZVS, including the position of calcifications.

## Conclusions

Understanding ZIKV pathways of entrance and spread in the embryonic CNS is fundamental for developing strategies for mitigating and preventing the devastating clinical outcomes. Here, we discussed how investigating CZVS can help us understand the natural history of the congenital infection. The first variable that we discussed was the time of infection. The developmental age at which ZIKV reaches the embryonic CNS is particularly relevant because not all the anatomical barriers and cell types are necessarily in place (Fig. 1). Research suggests that if ZIKV reaches the embryo before neural tube closure, the embryo will probably be non-viable. The second month of gestation is significant for the development of possible routes of CNS infection, because this is the stage at which the blood vessels enter the meninges and brain parenchyma (Marín-Padilla et al., 2012) and the cranial nerves form (Fig. 1A,D). Despite this basic understanding of congenital ZIKV susceptibility windows, our knowledge of the risks is far from complete. Adding to the challenge is the fact that the timing of infection of the mother may not necessarily coincide with the timing of vertical transmission to the fetus, as ZIKV can persist in the body long before the infection window for the fetal CNS opens.

The most common phenotype found in CZVS is malformation of the prosencephalon with preserved caudal regions (Fig. 2A). We propose that this phenotype is probably formed after cranial nerve infection, possibly by the contact of axon terminals with the developing digestive and respiratory systems. The amniotic fluid is in direct contact with these epithelia and can be the source of this fetal blood-independent pathway for ZIKV. Indeed, in a recent preprint, Koenig and collaborators inoculated rhesus monkeys with ZIKV at a late embryonic stage to describe the timing of infection of the tissues in the maternal–fetal interface (Koenig et al., 2023 preprint). Their results showed that ZIKV possibly reaches the fetus through a route that does not involve the placenta – by infecting the decidua and, later, the fetal membranes (Koenig et al., 2023 preprint). It is, therefore, possible that ZIKV can reach the embryo using pathways that do not involve the placenta and fetal blood infection. This prompts the question regarding which mechanisms and pathways govern the opening and closure of the infection window that gives ZIKV access to the embryonic CNS.

The timing of infection is also relevant for understanding the spread of ZIKV within the CNS. There are four possible routes of spread: (1) through the migration of infected cells (Fig. 3), (2) by spreading along neuronal projections, (3) by producing multiple leaks in the BBB, and (4) by infecting the cerebrospinal fluid (Fig. 1D; Kim et al., 2020). The CZVS phenotype for which malformation is restricted to the prosencephalon is likely best explained by intra-axonal viral spread, as this was experimentally observed in projections of the callosal and retinal neurons (van den Pol et al., 2017). Conversely, patients with cerebellar, brainstem and spinal cord atrophy probably experienced viral spread through a more efficient mechanism, such as through the cerebrospinal fluid and a generalized breakage of the BBB. As discussed above, ZIKV disrupts neuronal cell migration, meaning that migration of infected neurons is probably not a significant mechanism of viral spread in the context of CZVS.

It is essential to frame ZIKV tropism for specific cell types and how infection affects ontogenetic processes from the perspective of the time of infection. For example, ZIKV tropism for astrocytes is irrelevant for fetuses in which the virus cleared before the onset of astrogliogenesis, around the 22nd GW (de Azevedo et al., 2003). In another example, the neural tube comprises almost solely neural progenitors around the ventricles. Therefore, any calcifications forming due to ZIKV infection before neurogenesis begins will preferentially reside close to the ventricular surface, and calcifications in the cortical–subcortical junction should indicate a ZIKV infection in the 13-26th GW window, when massive neuronal migration occurs in the prosencephalon (Sidman and Rakic, 1973; Bayer and Altman, 2005).

Completing our knowledge on the mechanisms involved in ZIKV entrance and spread in the CNS will guide the strategies for mitigating clinical outcomes. For instance, if further research confirms that the amniotic fluid is a critical blood-independent source for ZIKV access to the embryo, approaches that aim at acquisition of immunity or at pharmacological interference should target this route.

Finally, reproducing CZVS phenotypes in experimental models will be necessary for gaining mechanistic insight into ZIKV-derived neurological conditions and for developing possible treatments. So far, there are no available animal models in which the prosencephalon is specifically malformed after congenital ZIKV infection. Therefore, it is likely that researchers using existing *in vivo* models are testing how ZIKV causes the worst phenotypes found in CZVS and are not able to uncover the pathological mechanisms responsible for the majority of cases.

CZVS is a devastating prenatal diagnosis. By integrating infection and developmental biology studies, our research community will advance our understanding of how ZIKV causes the broad spectrum of neurological phenotypes. However, to achieve meaningful clinical impact, this mechanistic research and its translation must consider all patients involved.
